# Arsenic Removal from Aqueous Solutions Using Fe_3_O_4_-HBC Composite: Effect of Calcination on Adsorbents Performance

**DOI:** 10.1371/journal.pone.0100704

**Published:** 2014-06-26

**Authors:** Shams Ali Baig, TianTian Sheng, Chen Sun, XiaoQin Xue, LiSha Tan, XinHua Xu

**Affiliations:** Department of Environmental Engineering, College of Environmental and Resource Sciences, Zhejiang University, Hangzhou, Zhejiang Province, China; RMIT University, Australia

## Abstract

The presence of elevated concentration of arsenic in water sources is considered to be health hazard globally. Calcination process is known to change the surface efficacy of the adsorbent. In current study, five adsorbent composites: uncalcined and calcined Fe_3_O_4_-HBC prepared at different temperatures (400°C and 1000°C) and environment (air and nitrogen) were investigated for the adsorptive removal of As(V) and As(III) from aqueous solutions determining the influence of solution's pH, contact time, temperature, arsenic concentration and phosphate anions. Characterizations from FTIR, XRD, HT-XRD, BET and SEM analyses revealed that the Fe_3_O_4_-HBC composite at higher calcination temperature under nitrogen formed a new product (fayalite, Fe_2_SiO_4_) via phase transformation. In aqueous medium, ligand exchange between arsenic and the effective sorbent site ( = FeOOH) was established from the release of hydroxyl group. Langmuir model suggested data of the five adsorbent composites follow the order: Fe_3_O_4_-HBC-1000°C(N_2_)>Fe_3_O_4_-HBC (uncalcined)>Fe_3_O_4_-HBC-400°C(N_2_)>Fe_3_O_4_-HBC-400°C(air)>Fe_3_O_4_-HBC-1000°C(air) and the maximum As(V) and As(III) adsorption capacities were found to be about 3.35 mg g^−1^ and 3.07 mg g^−1^, respectively. The adsorption of As(V) and As(III) remained stable in a wider pH range (4–10) using Fe_3_O_4_-HBC-1000°C(N_2_). Additionally, adsorption data fitted well in pseudo-second-order (*R* ^2^>0.99) rather than pseudo-first-order kinetics model. The adsorption of As(V) and As(III) onto adsorbent composites increase with increase in temperatures indicating that it is an endothermic process. Phosphate concentration (0.0l mM or higher) strongly inhibited As(V) and As(III) removal through the mechanism of competitive adsorption. This study suggests that the selective calcination process could be useful to improve the adsorbent efficiency for enhanced arsenic removal from contaminated water.

## Introduction

Arsenic is an ubiquitous element that has been recognized to exist in four oxidation states (−3, 0, +3 and +5). Owing to its multiple toxic effects, arsenic has received considerable attention globally [1]. Chronic exposures to arsenic may cause various toxic events in humans including cancers of liver, lung and bladder. Over 200 million people worldwide are reported to be exposed to high concentrations of arsenic via contaminated water creating a major global health concern [2], [3]. However, the World Health Organization (WHO) has reduced the recommended dose of arsenic in drinking water to 10 µg L^−1^ [3]. In the past, numerous arsenic remediation techniques have been documented [2], [4]–[9] including oxidation, coagulation and filtration, prcipitation and others [7], [10], [11]. Most of these techniques are technically more complex and normally associated with mixing of different chemicals which results in the generation of huge amounts of hazardous sludge for disposal [10]–[12].

One of the remediation techniques used against arsenic contamination is the adsorption technique which is considered to be highly effective for arsenic removal from water environments. The main advantages of this technique include its simple operation, economic reliability and least waste generation properties [8], [12]. Various adsorbents developed from different cost-effective sources have been successfully tested so far [2], [5], [8], [13]. Few highly adsorptive capacity nanostructure sorbents have been investigated in past years [14]; however, iron and its compounds, including iron oxides and iron hydroxides have shown more effective in arsenic removal [15]. In addition, from few of the recently reported studies, iron-oxide based composite sorbents were found to be most efficient as compared to other oxides or hydroxides [6], [16], [17]. For example, Luo et al. [6] utilized Fe_3_O_4_-reduced graphite oxide-MnO_2_ nanocomposite for arsenic removal. Similarly, Feng et al. [17] developed superparamagnetic high-surface-area Fe_3_O_4_ nanoparticles for the same purpose and their results also confirmed the efficiency enhancement of iron oxide based composite sorbents.

Honeycomb briquette cinders (HBC) are waste biomass materials produced from household-based cylindrical stoves in many countries including China. Recently, the scientific applicability and the attractive surface compositions of HBC for pollutant remediation have been extensively investigated [8], [18]. Previously, we had successfully employed iron amended HBC (Fe-HBC) as an adsorbent for arsenic removal in aqueous and column-based studies [5], [8]. It was found that the process of adsorption was significantly influenced by the adsorbent specific surface area and effective component compositions. To improve the surface properties of the absorbent, the process of calcination has been reported as one of the effective methods which can help to increase adsorbent hardness and decrease its water adhesion preventing the breakage of absorbent [19]. Generally, high temperature calcination process has shown to enhance adsorbent surface capacity and deformation of surface textural and mineralogical properties [20]. In contrast, Mahmood et al. [21] reported a decrease in arsenic adsorption efficiency by mixed oxide due to increased calcination temperature which resulted in decreasing the surface area of the adsorbent. However, the influence of calcination environments (air or nitrogen) and temperatures (lower or higher) on adsorbent composites for As(V) and As(III) removal has been rarely discussed in literature. Thereby, the optimal calcination temperature and environment for adsorbent development still needs to be elucidated.

In our current work, we evaluated the adsorption performance of calcined and uncalcined Fe_3_O_4_-HBC composites prepared at different temperatures (400°C and 1000°C) and environments (air and nitrogen) for the removal of As(V) and As(III) from aqueous solutions. The surface texture of Fe_3_O_4_-HBC composite and mineralogical variations were studied using XRD, HT-XRD, FTIR, BET and SEM techniques. The present study is an attempt to investigate the adsorption capacity of Fe_3_O_4_-HBC composites (uncalcined and calcined) for As(V) and As(III) from aqueous solutions which may help to elucidate the influence of solution pH, As(V) and As(III) concentration, calcination environment, contact time, temperature and the effects of phosphate anions.

## Materials and Methods

### Reagents

Chemicals including FeSO_4_.7H_2_O, FeCl_3_.6H_2_O, thiourea, L-ascorbic acid, potassium borohydride, hydrochloric acid solution, potassium di-hydrogen phosphate, Na_3_AsO_4_.12H_2_O and Na_3_AsO_3_ were all analytical grades and used without any purification. These chemicals were obtained from two dealers; i) Sinopharm Group Chemical Reagent Co., Ltd., China, and ii) Aladdin Chemistry Co., Ltd., Shanghai, China. As(V) and As(III) stock solutions (1000 mg L^−1^) were prepared by dissolving the weighted amount of Na_3_AsO_4_.12H_2_O and Na_3_AsO_3_, respectively, in the measured volume of de-ionized (DI) water.

### Preparation of raw and calcined Fe_3_O_4_-HBC composite

Wastes HBC biomass materials were obtained from a local restaurant at Hangzhou city in China and widely available in both cities and rural settlements. Initially, the brownish color HBC were crushed with a hammer and then sieved to obtain <0.5 mm granules. The sieved granules were washed several times with acid and DI water, and then dried in oven at 50°C for 24 h. The dried HBC<0.5 mm granules were purposively used to fabricate with Fe_3_O_4_.

The Fe_3_O_4_-HBC composite was synthesized by a modified *in situ* chemical co-precipitation method, which has been employed for Fe_3_O_4_ generation [16]. Briefly, 5 g of HBC was added to a 100 mL mixed solution of FeSO_4_.7H_2_O (2.78 g) and FeCl_3_.6H_2_O (5.40 g) under nitrogen flow. Additionally, the proportionate amount of ammonia was poured by drop wise addition and the reaction was held for 2 h at 60°C temperature. The fabricated Fe_3_O_4_-HBC composite was washed with DI water for several times to remove unnecessary ions. The final adsorbent was then dried in vacuum at 60°C for 24 h. Afterwards, the dried Fe_3_O_4_-HBC composite was calcined in high temperature furnace under different conditions for 2 h to obtain the other four adsorbent composites; 1) uncalcined Fe_3_O_4_-HBC composite, 2) calcined at 400°C under air (Fe_3_O_4_-HBC-400°C(air)), 3) calcined at 400°C under nitrogen (Fe_3_O_4_-HBC-400°C(N_2_)), 4) calcined at 1000°C under air (Fe_3_O_4_-HBC-1000°C(air)) and 5) calcined at 1000°C under nitrogen (Fe_3_O_4_-HBC-1000°C (N_2_)).

### Batch adsorption experiments

In this study, the effects of initial pH, equilibrium time, temperature, As(V) or As(III) concentration, kinetic and adsorption isotherms were investigated. Batch adsorption experiments were performed in a series of 100 mL glass conical flasks containing As(V) or As(III) aqueous solutions with 0.02 g calcined and uncalcined Fe_3_O_4_-HBC composite at 25±0.8°C. For adsorption experiments, 0.02 g calcined and uncalcined Fe_3_O_4_-HBC composite adsorbents were suspended in As(V) or As(III) aqueous medium with known concentrations and shaken with a speed of 150 rpm for 14 h. Afterwards, the suspensions were filtered using 0.45 µm filter membranes and the filtrate was subjected to analysis for the remaining total arsenic concentration. The adsorption capacity (q_e_) and removal efficiency (RE (%)) were calculated using equations (1) and (2):
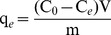
(1)

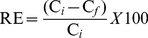
(2)where C_0_ and C_i_ represent the initial As(V) or As(III) concentration, and C_e_ and C_f_ are the equilibrium or final concentration of the respective arsenic species in aqueous solution. V and m represent the volume of the aqueous solution and adsorbent mass, respectively.

Experiments for the kinetics of five Fe_3_O_4_-HBC adsorbent composites were performed by adding 0.02 g of adsorbent into 100 mL arsenic solution with the initial concentration of 100 µg L^−1^ at pH 7.0. Samples were taken out at certain intervals using syringes and filtered. The effects of various pH values (pH 2–12) on As(V) and As(III) adsorption were performed by adjusting the aqueous solution pH using HCl and NaOH in 14 h contact time. Experiments for the equilibrium adsorption isotherms were performed with different initial arsenic concentrations containing 0.02 g of adsorbent at pH 7.0. The influence of temperature on As(V) and As(III) adsorption was investigated by varying the aqueous solution temperatures (25°C, 35°C and 45°C). Moreover, the effects of different PO_4_^3−^ concentrations on arsenic removal were investigated by adding different concentrations (0–1 mM) of phosphate into arsenic containing solutions. Each sample was tested in triplicates and the mean values were expressed with standard error.

### Adsorbents characterization

Fourier transform infrared spectroscopy (FTIR) of calcined and uncalcined adsorbent composites was recorded at 4000 and 400 cm^−1^ wavenumber (IR Prestige-21, Japan). Scanning electron micrograph (SEM) (Hitachi S-3000N, Japan) was used to analyze the morphological structures of Fe_3_O_4_-HBC composite before and after calcination at different temperatures and environments. X-ray diffraction (XRD) analysis (X'pert PRO analytical B.V., Netherlands) of the adsorbent composites was performed. In order to investigate the phase transformation of the adsorbent composite (Fe_3_O_4_-HBC-1000°C(N_2_)) *in situ* high temperature-X-ray diffraction (HT-XRD) (PANalytical, X'Pert PRO, Philips, Almelo, Netherlands) was performed at the heating rate of 10°C min^−1^ under nitrogen environment and HT-XRD data were taken after heating at 1000°C. Brunauer–Emmett–Teller (BET) surface area of the samples was measured using BET surface analyzer (ASAP 2050, Micrometrics, Beijing, China).

### Analytical methods

Atomic fluorescence spectroscopy (model AFS-230E, Beijing Kechuang Haiguang Instrument Company, China) was used for the determination of total arsenic concentration [5], [8]. The total arsenic detection limits and repeatability in AFS were 1 µg L^−1^ and 10%, respectively. A digital pH meter (Mettler Toledo SG2, Shangai, China) was used to measure solution pH. High temperature furnace (model: GSL1500X Hefei, China) with the heating rate of 10°C min^−1^ was used to calcine Fe_3_O_4_-HBC composite at 400°C and 1000°C under nitrogen flow and in static air (200 cc min^−1^).

## Results and Discussion

### Characterization of adsorbent composites

**Figure 1:**
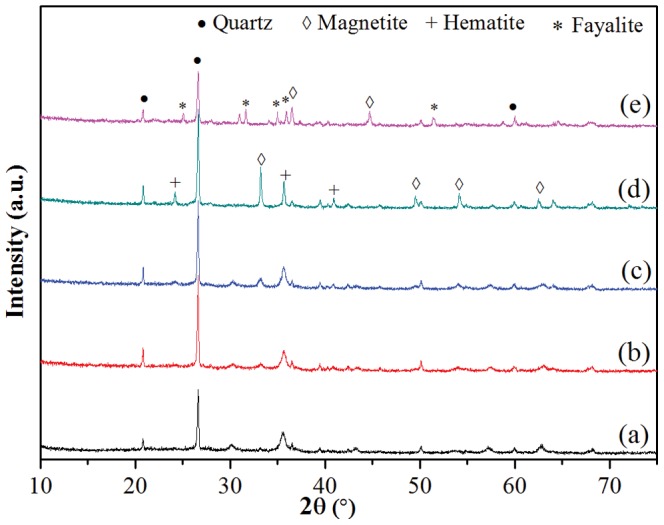
XRD pattern of five adsorbent composites: (a) uncalcined Fe_3_O_4_-HBC composite, (b) 400°C under air, (c) 400°C under nitrogen, (d) 1000°C under air, and (e) 1000°C under nitrogen. Different XRD peaks are also marked.

The XRD patterns of the five Fe_3_O_4_-HBC adsorbent composites are presented in Fig. 1. The XRD peaks obtained revealed that quartz and magnetite were the major phases in uncalcined Fe_3_O_4_-HBC composite (Fig. 1a) and consistent with the corresponding values [8], [18]. It was observed that with the increase in calcination temperature, there was an increase in the intensities of quartz and magnetite as shown in Fig 1b–d. Major peaks of quartz and magnetite were observed to appear at 2*θ* (20.8°, 26.6°, 39.4°, 50.1° and 59.6°) and 2*θ* (24.1°, 33.1°, 54.1° and 62.4°), respectively. During high temperature calcination under air some of the magnetite was oxidized into hematite and the new peaks appeared were marked “+” in the XRD pattern (Fig. 1d). Higher calcination temperature exhibited a significant effect on phase transformation, especially at 1000°C under nitrogen. Thus, most of the quartz and magnetite phases disappeared and formed a new product, fayalite (Fe_2_SiO_4_) as a major phase with the peaks appearing at 2*θ* (25.0°, 31.5°, 34.9°, 35.3°, and 51.1°) (Fig. 1e). High temperature calcination of quartz and magnetite under nitrogen environment has been recognized to favor the formation of fayalite [22], [23]. HT-XRD analysis of the adsorbent composite (Fe_3_O_4_-HBC-1000°C(N_2_)) also confirmed the formation of fayalite (Fig. S1) and there was an increase in the relative intensity of fayalite recorded at 1000°C under nitrogen environment as compared to XRD patterns (Fig. 1e). In such anhydrous environment, oxygen fugacity was found to be very low to maintain the stability of ferrous iron to react with silicate. In contrast, high temperature calcination under air can produce magnetite and silicate from fayalite [24]. From this study it revealed that the high temperature calcination under controlled environment would only guarantee phase transformation, as shown in Fig. 1e. But the low temperature (400°C) calcination under nitrogen environment cannot favor the formation of fayalite (Fig. 1c).

**Figure 2:**
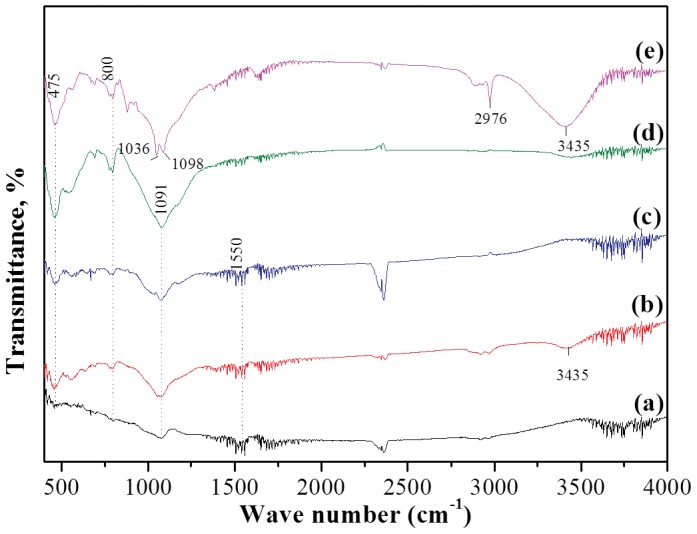
FTIR spectra of (a) uncalcined Fe_3_O_4_-HBC composite, (b) 400°C under air, (c) 400°C under nitrogen, (d) 1000°C under air, (e) 1000°C under nitrogen.

The presence of different functional groups on the adsorbent was analyzed by FTIR analysis (Fig. 2). The appearance of band at 475 cm^−1^ was attributed to Fe-O vibration, which was observed to become stronger with the increase in the calcination temperature. However, H-O-H bonding in water was assumed to be responsible for the appearance of band at 1550 cm^−1^. Moreover, a strong band was found to appear at 1091 cm^−1^ in the FTIR spectra (Fig. 2a–d) representing Si-O-Si stretching mode. The band observed around 1036 cm^−1^ and 1098 cm^−1^ in Fe_3_O_4_-HBC-1000°C(N_2_) confirmed the fayalite formation [23]. Sharp peak and broader absorption bands observed at 2976 cm^−1^ and 3435 cm^−1^ in Fe_3_O_4_-HBC-1000°C(N_2_) could be assigned to C-H stretching vibration and OH^−^ vibrational modes, respectively. Moreover, C-H stretching vibration band in the same adsorbent reflected the presence of absorbing carbon [25].

**Figure 3:**
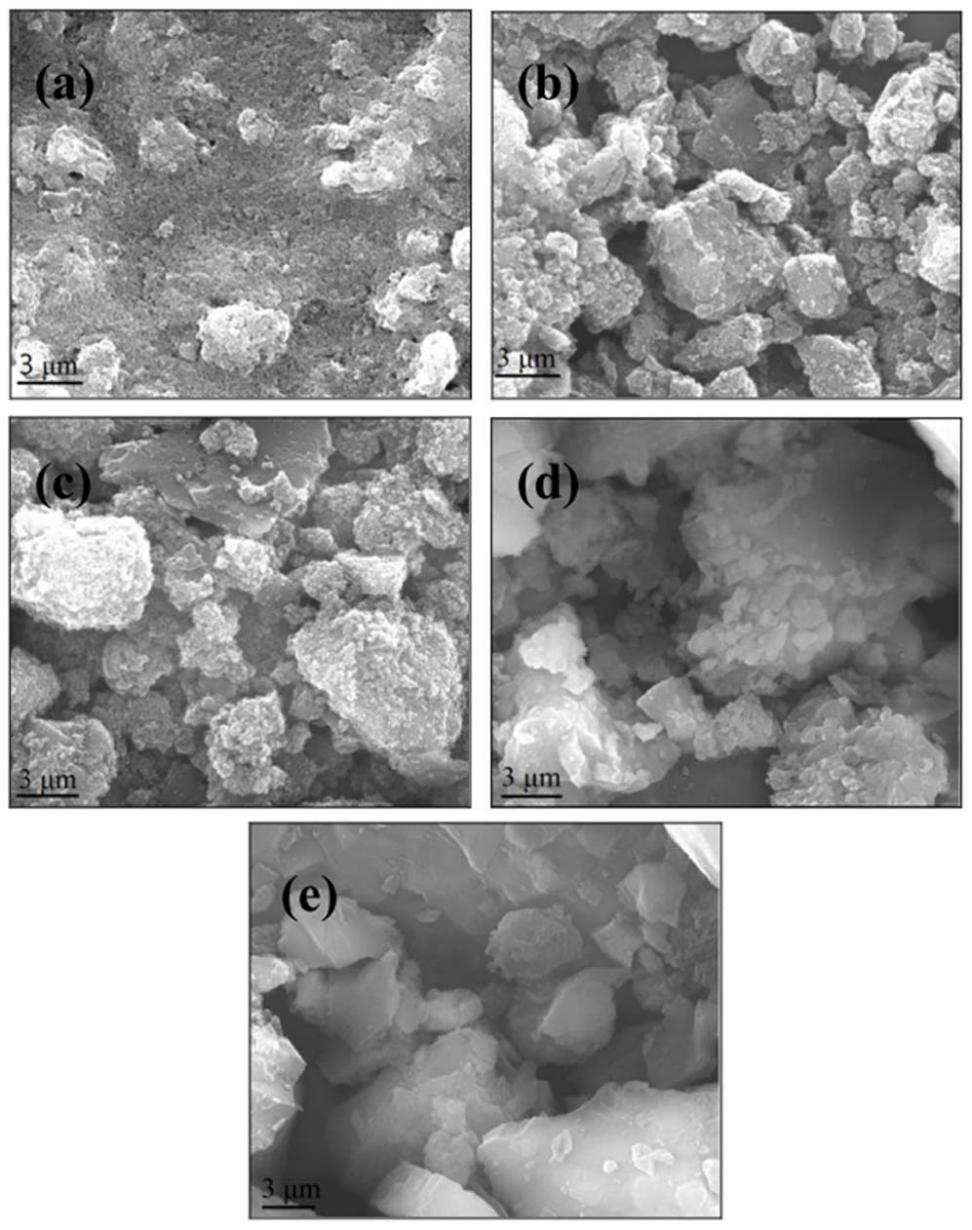
SEM images of (a) uncalcined Fe_3_O_4_-HBC composite, (b) 400°C under air, (c) 400°C under nitrogen, (d) 1000°C under air, (e) 1000°C under nitrogen.

Increase in calcination temperature resulted in coarsening of particles due to sintering processes [20]. According to SEM images shown in Fig. 3a–e, it can be seen that the calcined Fe_3_O_4_-HBC composite at 400°C and 1000°C, showed polyhedral and heterogeneous particles size distributions. But there were some morphological and mineralogical differences observed in adsorbents calcined under air and nitrogen flow. It has been suggested that elevated calcination temperature can remove water and organics from the adsorbent and tends to sinter and crystallize, which cause to decrease the surface area [26]. In our study, the surface area of all the calcined adsorbents were drastically decreased from 33.05 (m^2^ g^−1^) of Fe_3_O_4_-HBC (uncalcined) to 0.04, 0.03, 0.95 and 6.03 (m^2^ g^−1^) of Fe_3_O_4_-HBC-400°C(N_2_), Fe_3_O_4_-HBC-400°C(air), Fe_3_O_4_-HBC-1000°C(air), Fe_3_O_4_-HBC-1000°C(N_2_), respectively, as shown in Table 1. Additionally, the mean pore diameter of the adsorbents also greatly varied from each other (Table 1).

**Table 1:** Textural characteristics of uncalcined and calcined Fe_3_O_4_-HBC composites.

Adsorbent	Surface area (m^2^ g^−1^)	Total pore volume V_p_ (cm^3^ g^−1^)	Mean pore diameter (nm)
Fe_3_O_4_-HBC uncalcined	33.05	0.0966	13.35
400°C under air	0.04	0.0027	187.54
400°C under nitrogen	0.03	0.0018	229.79
1000°C under air	0.95	0.0851	31.73
1000°C under nitrogen	6.03	0.0080	15.01

Generally, sintering is known to start between 100°C and 300°C, but the most extensive crystallization processes could take place in between 500–700°C, where the porosity and surface area may markedly decrease [20]. Similarly, Kurtoglu et al. [25] found that stronger and clear bands were only appeared at a maximum calcination temperature (i.e. 1000°C). In this study, calcination at 1000°C under nitrogen resulted in the formation of a new iron silicate product (fayalite, Fe_2_SiO_4_) with an average pore diameter of 15.01 nm (Fig. 3e and Table 1). Moreover, the calcined products formed at 400°C under air and nitrogen nearly showed the same mean pore diameter and BET surface area (Table 1). But the adsorbent calcined at 1000°C showed significant annealing or textural modification from 400°C to 1000°C after 2 h of calcination. Nitrogen-calcined composite showed better phase transformation at higher temperature, as shown in XRD and HT-XRD patterns (Fig. 1e and Fig. S1). In contrast, insignificant annealing was also reported even at 800°C in run products (fayalite, magnetite and quartz) [26].

### Calcination environments and Fe_3_O_4_-HBC composite products

Magnetite and quartz were the major phases observed in the adsorbent composite, as revealed in XRD patterns (Fig. 1a). High temperature calcination (1000°C) under nitrogen generated a new iron silicate phase (fayalite, Fe_2_SiO_4_) (Fig. 1e and Fig. S1). Fayalite formation can be written, as Eq. (3) [22], [23]. 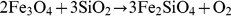
(3)


Fayalite is known to form in an environment where the oxygen fugacity is low enough to maintain the stability of ferrous iron [22]. Fayalite is the reaction product of magnetite and quartz under the reducing environment with low water content. In this study, the oven-dried Fe_3_O_4_-HBC composite was calcined and amount of water content was negligible. Fe_3_O_4_-HBC composite calcined at higher temperature under nitrogen contains significant amount of carbon from the carbonization of organic precursor, which can be reflected by C-H stretching vibration mode observed at 2976 cm^−1^ in FTIR spectra (Fig. 2e). No C-H stretching vibration mode was recorded in the adsorbent calcined at high temperature under air indicating complete combustion of organic materials (Fig. 2d).

High temperature calcination under air signified the role of oxic-environment, which further enhanced magnetite and quartz contents in the adsorbent (Fig. 1d). Similarly, Michel et al. [24] reported that at high calcination temperature under air, the fayalite is known to be oxidized and form quartz and magnetite, according to the following reaction (Eq. (4)). 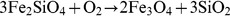
(4)


Correspondingly, Shahar et al. [26] reported that in oxygen fugacity buffer, a stable assemblage comprising of quartz-fayalite-magnetite (QFM) could be formed. But the fayalite stability analysis under different calcination environments was not within the scope of this study. However, further studies are required to measure the oxygen fugacity at elevated calcination temperature under air and its reaction with arsenic in contaminated water.

### Effect of solution pH

**Figure 4:**
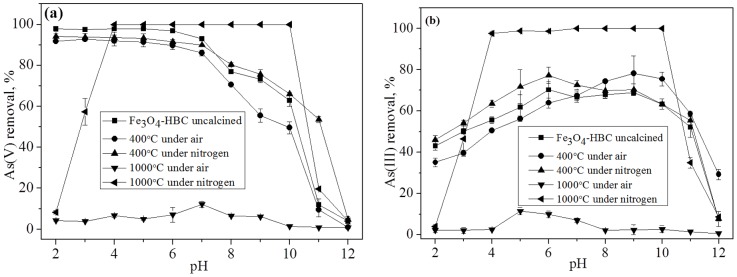
Effects of initial solution pH on the removal of As(V) (a) and As(III) (b) (Experiment conditions: adsorbent dose  = 0.02 g 100 mL^−1^, temperature  = 25±0.8°C, agitation speed  = 150 rpm, initial concentration  = 100 µg L^−1^, contact time  = 14 h).

The effects of solution pH on As(V) and As(III) adsorption by calcined and uncalcined adsorbents were investigated at different pH values (2–12) (Fig. 4a and b). Arsenic adsorption is known to be pH-dependent, moreover, the surface groups of the adsorbent are also considered vulnerable to be protonated and deprotonated [9], [16]. The removal efficiency of As(V) using uncalcined and calcined adsorbent composites at 400°C in both air and nitrogen, respectively, was decreased with the increase in the initial pH (Fig. 4a). Higher adsorption of arsenic at lower pH may be due to the electrostatic attraction of the positively charge adsorbent sites and the negatively charged H_2_AsO_4_^−^ species. Generally, As(V) is known to exist in negative ionic form under most of the pH conditions [16]. Correspondingly, we can assume that at low pH the adsorbent composites should behave as weak acid and form positive surface site for anionic arsenic adsorption by forming inner-sphere surface complexes. However, As(V) removal efficiency was observed to drastically decrease above pH 7, which is in agreement with previous studies [6]. However, Fe_3_O_4_-HBC-1000°C(N_2_) showed the highest As(V) adsorption at wider pH range (4–10). In aqueous medium, Fe_2_SiO_4_ showed to form a highly reactive iron species ( = FeOOH) [9], [23] and that bound to As(V) and As(III) by ligand exchanges. The occurrence of ligand exchange between As(V) and As(III) with adsorbent effective site ( = FeOOH) suggested the completion of sorption process by forming inner-sphere surface complexes at the solid-water interface, as written in Eqs. (5), (6) and (7).
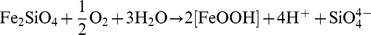
(5)

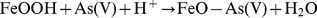
(6)

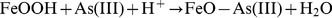
(7)


Fe_3_O_4_-HBC-1000°C(air) showed to have the lowest efficiency (<10%) at the studied pH range (2–12) (Fig. 4a). Variations of the effective surface component at high calcination temperature enabled the adsorbent as an efficient agent for treating arsenic contaminated water. The removal efficiency of As(III) reached to nearly 70% at pH 6 for Fe_3_O_4_-HBC-400°C(N_2_) and was observed to decrease at both low and high pH values (Fig. 4b), as reported earlier [27]. Generally, As(III) exists as neutral species at pH<9.2 that can be deprotonated to form more negatively charged anions (H_2_AsO_3_^−^) [7]. The reduction in arsenic removal with the increase in the solution pH could be attributed to the electrostatic repulsion of the negatively charged adsorbent surface from the anionic H_2_AsO_4_^−^, HAsO_4_^2−^ and H_2_AsO_3_^−^ species [7], [21]. Consequently, the combined effects of enhanced electrostatic repulsion and the hydroxyl ion competition can be accounted responsible for the declined arsenic removal in alkaline aqueous conditions. However, the efficient removal of As(V) and As(III) by Fe_3_O_4_-HBC-1000°C(N_2_) might be the result of the surface component (SiO_4_^4−^), which further resulted in enhanced adsorption within a wider pH range (pH 4–10). The final solution pH in case of adsorbent (Fe_3_O_4_-HBC-1000°C(N_2_)) showed considerable increase at equilibrium for both As(V) and As(III) as compared to other adsorbent composites (Fig. S2).

### Effect of contact time and adsorption kinetics

**Figure 5:**
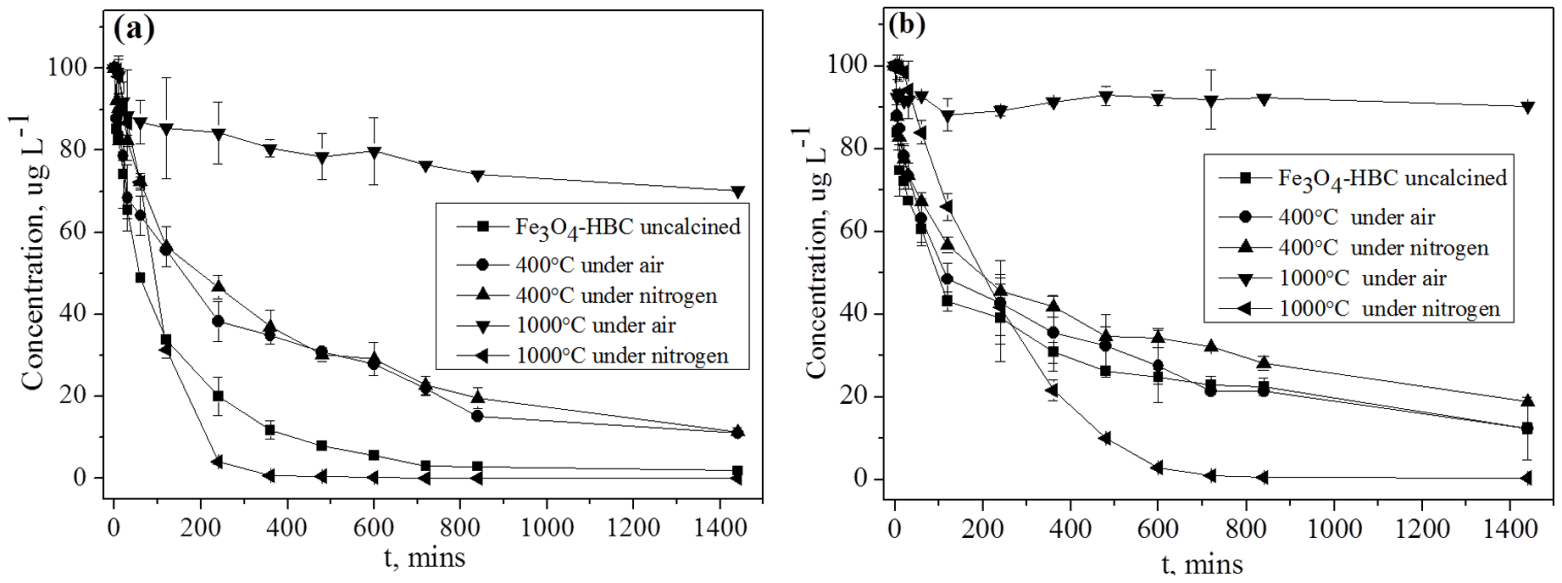
The effect of contact time on the removal of As(V) (a) and As(III) (b) by the uncalcined and calcined Fe_3_O_4_-HBC composite (Experiment conditions: adsorbent dose  = 0.02 g 100 mL^−1^, temperature  = 25±0.8°C, agitation speed  = 150 rpm, initial concentration  = 100 µg L^−1^, contact time  = 14 h).

Generally, adsorption onto the adsorbent from aqueous solution involves three steps, namely; 1) transport into the exterior surface (film diffusion), 2) transport into the pore and surface diffusion (intraparticle diffusion), and 3) adsorption onto the adsorbent surface from the bulk phase. Hence, the overall adsorption rate is determined by the slowest adsorption step involved [28]. Fig. 5a and b presents As(V) and As(III) removal using five adsorbent composites along with contact times. All the adsorbents except Fe_3_O_4_-HBC-1000°C(air) were found significantly effective for the removal of both As(V) and As(III), especially Fe_3_O_4_-HBC-1000°C(N_2_). But Fe_3_O_4_-HBC (uncalcined), Fe_3_O_4_-HBC-400°C(N_2_) and Fe_3_O_4_-HBC-400°C(air) demonstrated over 80% removal efficiencies for both As(V) and As(III). Rapid reduction was recorded in the first 4 h for both As(V) and As(III), which subsequently slowed down and reached to equilibrium within 14 h contact time. This could be attributed to the phenomenon that higher concentration gradient and more adsorptive sites favor fast adsorption initially. However, when the adsorption experiments were extended up to 24 h, no significant adsorption was observed (Fig. 5a and b).

Adsorption kinetics depends on the interaction of adsorbate-adsorbent in aqueous adsorption system and plays an important role in water purification measures. Reaction rate and adsorption mechanism are the two key elements in adsorption process. The uptake of solute rate determines the retention time required to complete the adsorption process, which can be enumerated from kinetic studies. Numerous models can be employed to express the solute adsorption onto an adsorbent [29], [30]. In order to investigate the adsorption mechanism, characteristic constants, and the solid phase adsorption pseudo-first order (PFO) [29] and pseudo-second order (PSO) kinetics models [30] can be used. Thus, in this study PFO and PSO models were introduced to fit the adsorption kinetics of As(V) and As(III) onto uncalcined and calcined Fe_3_O_4_-HBC composites (Fig. S3). The PFO and PSO adsorption kinetics equations are presented in Eqs. (8) and (9):
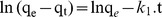
(8)

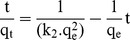
(9)where q_e_ and q_t_ (mg g^−1^) are the amount of solute adsorbed per unit mass of adsorbent at equilibrium time t (min), *k*_1_ (min^−1^) is the PFO adsorption rate constant, *k*_2_ (g mg^−1^ min^−1^) is the PSO adsorption rate constant and *k*_2_.q_e_^2^ (mg g^−1^ min^−1^) is the PSO adsorption rate at t = 0 (min). The parameters of kinetics model obtained from PFO and PSO calculations (Fig. S3) and experimental data are summarized in Table 2. According to Table 2, the adsorption of both As(V) and As(III) fitted well in PSO kinetics model (*R* ^2^>0.99) rather than PFO kinetics model (*R* ^2^>0.91) for all the adsorbents. Furthermore, the adsorption capacity (q_t_) at any time t (min) of As(V) was found to be greater than that of As(III). The adsorption equilibrium amount q_e_ (628 µg g^−1^) predicted in the PSO kinetics model was observed to be similar to the experimental value (552 µg g^−1^) (Table 2). Kinetic data revealed that the mechanisms for both As(V) and As(III) removal were complex and most likely all of the three aforementioned steps might be involved in the adsorption of arsenic onto the adsorbent from aqueous solutions. The fitted data in PSO kinetics model are assumed to follow chemisorption phenomenon [28]. However, with rate limited chemisorptions; the precipitation and inner-sphere complexation are known to be involved between the adsorbate and adsorbent.

**Table 2:** Comparisons of pseudo-first and pseudo-second-order models calculated reaction constants for uncalcined and calcined Fe_3_O_4_-HBC composites with the experimental data.

Adsorbent	Arsenic species	Pseudo-first order kinetic model			Pseudo-second order kinetic model			Experimental data q_e_(µg g^−1^)
		k_1_(min^−1^)	q_e_(µg g^−1^)	*R* ^2^	k_2_ (g mg^−1^ min^−1^)	q_e_(µg g^−1^)	*R* ^2^	
Fe_3_O_4_-HBC uncalcined	As(V)	0.0023	13.0	0.9808	0.0428	564.1	0.9984	547.4
400°C under air	As(V)	0.0008	12.0	0.9233	0.0117	387.2	0.9930	404.2
400°C under nitrogen	As(V)	0.0011	13.0	0.9796	0.1978	476.0	0.9900	500.0
1000°C under air	As(V)	0.0008	08.0	0.8985	0.0794	132.4	0.9809	150.2
1000°C under nitrogen	As(V)	0.0049	15.0	0.9889	0.0182	628.1	0.9917	552.1
Fe_3_O_4_-HBC uncalcined	As(III)	0.0010	11.0	0.9816	0.0461	371.4	0.9964	401.0
400°C under air	As(III)	0.0011	12.0	0.9271	0.0255	390.4	0.9924	401.2
400°C under nitrogen	As(III)	0.0009	11.0	0.9657	0.0329	340.0	0.9939	366.2
1000°C under air	As(III)	0.0004	02.0	0.1832	15.755	6.1	0.9695	9.76
1000°C under nitrogen	As(III)	0.0032	18.0	0.9817	0.0034	806.4	0.9960	560.2

### Effect of temperature

To study the effect of temperature on the adsorption capacity of the adsorbent composites is very important for designing a sustainable adsorption system. Our results demonstrated that with the increase in temperature from 25°C to 45°C, the adsorption capacities of the adsorbent composites were slightly increased (Table S1). This showed that the adsorption process is an endothermic and controlled by the intraparticle and pore diffusion [3], [9]. The mobility of adsorbent ions increases with the increase of the aqueous temperature, thus increasing the adsorptive capacity of the adsorbent composites. Similarly, the adsorption capacity of magnetite-graphene hybrids for arsenic was found to be increased from 10°C to 30°C [27].

### Adsorption isotherm studies

**Figure 6:**
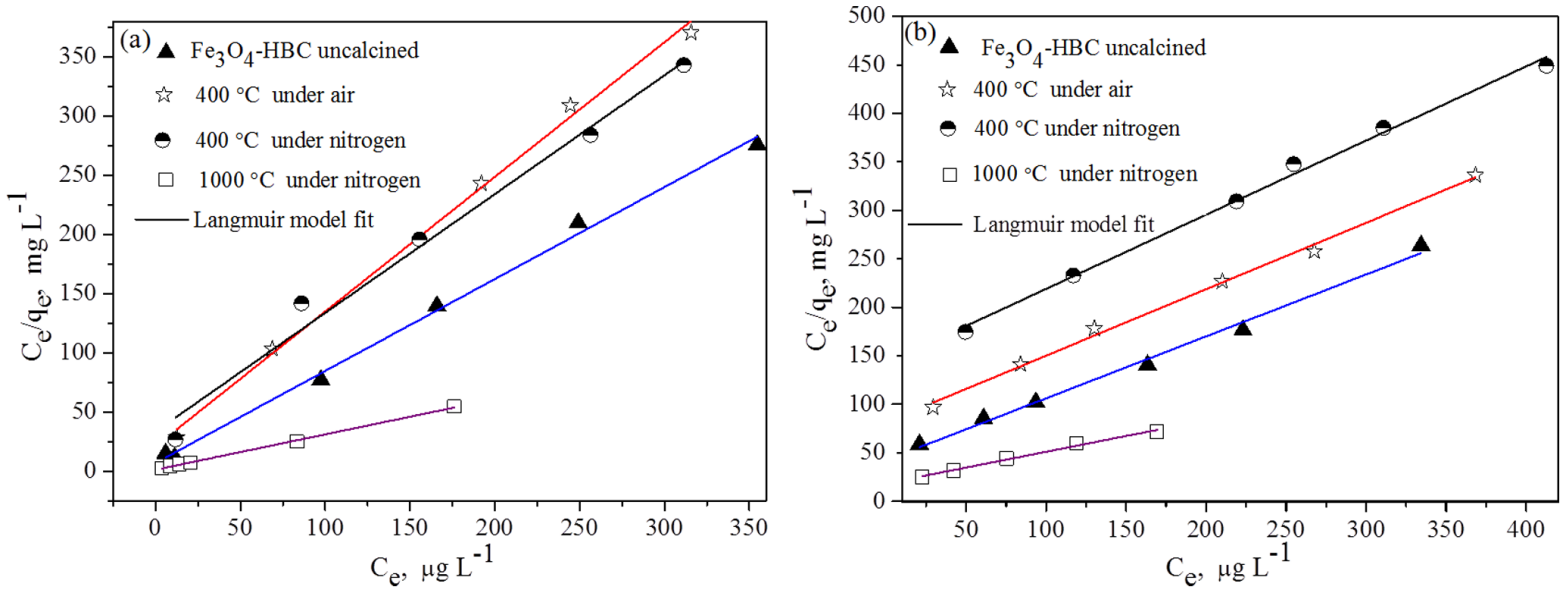
Adsorption isotherm parameters for As(V) (a) and As(III) (b) adsorption onto calcined and uncalcined Fe_3_O_4_-HBC composite at pH 7.0 and temperature (25±0.8°C).

To help optimize the process for removal of arsenic, it is important to understand arsenic distribution between phases via analyzing the equilibrium data. In this study, equilibrium adsorption isotherm studies were performed for four adsorbent composites: Fe_3_O_4_-HBC (uncalcined), Fe_3_O_4_-HBC-400°C(N_2_), Fe_3_O_4_-HBC-400°C(air) and Fe_3_O_4_-HBC-1000°C(N_2_). Langmuir model is valid for a monolayer sorption mechanism with homogeneous sorption energies [31]. Monolayer adsorption onto the adsorbent surface has finite number of similar sites. Generally, it is assumed that further metal ions adsorption could not take place once the surface is saturated from that ions [32]. The Langmuir model [31] can be expressed as Eq. (10). 
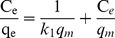
(10)where C_e_ and q_e_ are arsenic equilibrium concentrations and adsorption capacity (mg g^−1^), respectively, based on the dry weight of the adsorbents. *k_1_* is the Langmuir adsorption equilibrium constant (L mg^−1^) and *q_m_* is the maximum adsorption capacity (mg g^−1^). The plot between C_e_/q_e_ (along y-axis) and C_e_ (along x-axis) at different initial As(V) and As(III) concentrations showed to generate straight lines (Fig. 6a and b), which revealed that the adsorption process of As ions onto the adsorbent composites followed the Langmuir adsorption Eq. (10). However, more straight lines (*R* ^2^>0.99) were observed for As(V) as compared to As(III) (Table 3). This variation in adsorption isotherm studies might be the differences in adsorption free energies of As(V) and As(III) [16], [32], [33]. Generally, As(V) species (H_2_AsO_4_^−^) is predominantly available in the studied aqueous solution pH (∼7.0) which has lower free adsorption energy as compared to As(III) species (H_3_AsO_3_). Thus, the adsorption capacity of As(V) was found higher than that of As(III) which is in agreement with previously reported studies [25], [27], [34], but disgreement with study reported by Luo et al. [6]. The maximum calculated adsorption for As(V) followed the order: Fe_3_O_4_-HBC-1000°C(N_2_)>Fe_3_O_4_-HBC (uncalcined)>Fe_3_O_4_-HBC-400°C(N_2_)>Fe_3_O_4_-HBC-400°C(air). The maximum adsorption capacities (*q_m_*) of Fe_3_O_4_-HBC-1000°C(N_2_) for As(V) and As(III) were recorded to be 3.37 mg g^−1^ and 3.07 mg g^−1^, respectively (Table 3). Hence, among the other adsorbents presented in Table 4, Fe_3_O_4_-HBC-1000°C(N_2_) was found highly effective than that of iron-amended adsorbents, but less efficient than that of Fe_3_O_4_-nanocomposites.

**Table 3:** Langmuir model parameters for the adsorption of As(V) and As(III) on Fe_3_O_4_-HBC composite at ambient temperature (25±0.8°C).

Adsorbent	Arsenic species	Langmuir model parameters		
		q_m_ (mg g^−1^)	*k_1_* (L mg^−1^)	*R* ^2^
Fe_3_O_4_-HBC uncalcined	As(V)	1.288	109.35	0.9754
400°C under air	As(V)	0.880	54.13	0.9656
400°C under nitrogen	As(V)	0.998	30.06	0.9801
1000°C under nitrogen	As(V)	3.355	254.56	0.9988
Fe_3_O_4_-HBC uncalcined	As(III)	1.566	14.96	0.9811
400°C under air	As(III)	1.460	8.38	0.9753
400°C under nitrogen	As(III)	1.308	5.36	0.9740
1000°C under nitrogen	As(III)	3.071	17.57	0.9984

**Table 4:** Comparison of the adsorption capacities of different adsorbents for As(V) and As(III).

Adsorbent	pH	Temperature(°C)	Arsenic removal (mg g^−1^)		Reference
			As(V)	As(III)	
Fe-HBC	7.5	23±2	0.9	-	[8]
Magnetic wheat straw (MWS)	7.0	30	8.02	3.8	[16]
Ascorbic acid-coated-Fe_3_O_4_ nanoparticles	7.0	25	16	46	[17]
Biochar/γ-F e_2_O_3_ composite	7.0	22±0.5	3.1	-	[34]
Natural iron ores	6.5	25	0.4	-	[36]
Fe_3_O_4_-HBC composite	7.0	25±08	3.35	3.07	This study

In Langmuir model, the isotherm curve is considered to estimate the sorption process in term of “favorable” and “unfavorable” [8]. Thus, the constant *k*_1_ is related to the equilibrium parameter; *R*_L_, which can be defined as [35]: 
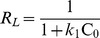
(11)where C_0_ (mg L^−1^) is the initial concentration of As(V) and As(III). The values of *R*_L_ for As(V) and As(III) within the studied C_0_ range were observed to be 0.061–0.332 and 0.042–0.651, respectively. Therefore, the calculated values of “*R*_L_” were found to be in between 0 and 1 indicating the adsorption of As(V) and As(III)onto uncalcined and calcined Fe_3_O_4_-HBC composite is a favorable process.

### Effects of phosphate anion on arsenic removal

**Figure 7:**
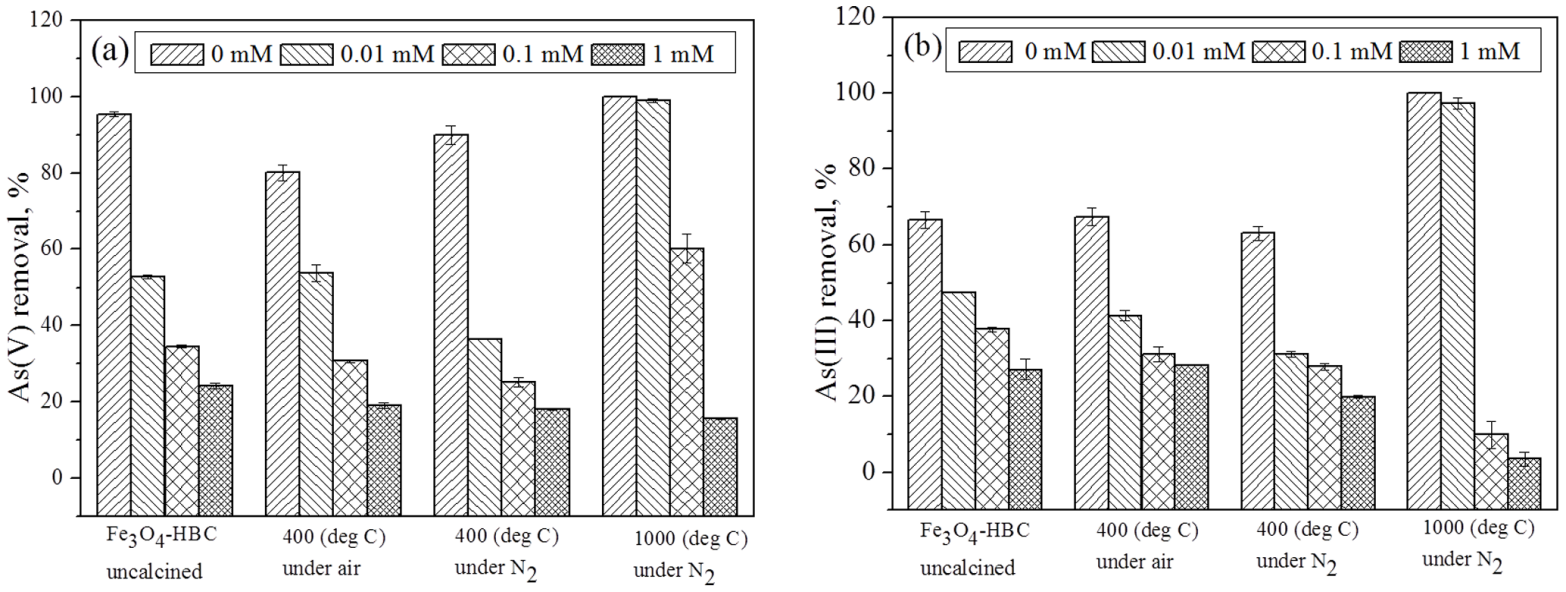
Effect of phosphate concentrations (0–1 mM) on the adsorption of As(V) (a) and As(III) (b) using uncalcined and calcined Fe_3_O_4_-HBC composite. Experiment conditions: adsorbent dose  = 0.02 g 100 mL^−1^, temperature  = 25±0.8°C, agitation speed  = 150 rpm, initial concentration  = 100 µg L^−1^, contact time  = 14 h).

The presence of phosphate in water is reported to significantly lower the ability of iron containing adsorbent to remove arsenic by adsorption [33]. Fig. 7a and b presents the interference study of different PO_4_^3−^ strengths (0–1 mM) on As(V) and As(III) adsorption from aqueous solutions using four composite adsorbents: Fe_3_O_4_-HBC (uncalcined), Fe_3_O_4_-HBC-400°C(N_2_), Fe_3_O_4_-HBC-400°C(air) and Fe_3_O_4_-HBC-1000°C(N_2_). Results showed that both As(V) and As(III) removal efficiencies decreased while increasing the phosphate concentration, which is in agreement with the studies reported by [33], [36]. For example, Chowdhury and Yanful [33] trailed natural groundwater containing 5 mg L^−1^ phosphate and 1.13 mg L^−1^ arsenic, where only half of the arsenic was removed by mixed magnetite-maghemite nanoparticles. Phosphate is recognized for its interference in the binding of arsenic to various materials, similarly, in our study we found that phosphate concentration (0.01 mM or above) has a significant effect on the removal of both; As(V) and A(III). However, initially at lower concentration (∼0.01 mM) of PO_4_^3−^, the binding of arsenic to Fe_3_O_4_-HBC1000°C(N_2_) remained to be unaffected. This clearly suggests that the low concentration of phosphate has weaker attraction due to the presence of surface component (SiO_4_^4−^), as written in Eq. (5). Moreover, 0.1 mM PO_4_^3−^ concentration exhibited significant As(V) removal efficiency (above 60%); however, it showed to remove only 10% of As(III) at the same PO_4_^3−^ concentration level (Fig. 7a and b).

Higher phosphate concentration has known to inhibit arsenic removal through the mechanisms of charged diffusion and competitive adsorption [9], [37]. In addition, formation of more stable phosphate complexes (Fe(H_2_PO_4_)_3_) with Fe(III) has been suggested another possible adsorption mechanism [38]. On the other hand, under same conditions, As(III) affinity towards uncalcined and calcined composite adsorbents was found to be weaker as compared to that of As(V) and phosphate. Consequently, the removal efficiency of As(III) was lowered and consistent with the findings reported in previous studies [9], [39]. Moreover, our work showed the probable effect of solution pH, As(V) and As(III) concentrations, calcination environment, temperature, contact time and the effects of phosphate anions on As(V) and As(III) removal.

### Removal mechanism of arsenic

**Figure 8:**
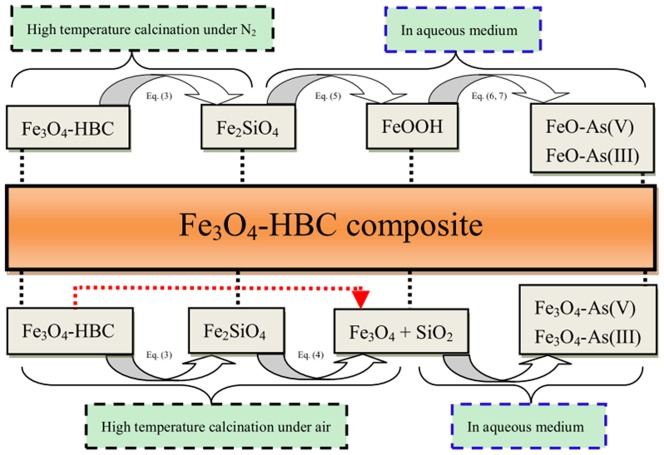
The proposed removal mechanism for As(V) and As(III) in aqueous system.

Fig. 8 presents the schematic possible mechanism of As(V) and As(III) adsorption from aqueous system. So far, various probable reaction pathways have been proposed for the adsorption mechanism of arsenic using different adsorbents; however, the mechanism of arsenic removal from aqueous solution is still partially understood [40]. In this study, a new iron silicate phase (fayalite, Fe_2_SiO_4_) was formed due to high temperature calcination under nitrogen environment, as discussed previously. The elevated calcination temperature is known to decrease the surface area and increase the particle size, which can ultimately decrease the removal performance [20], [21]. The substantial changes in the surface area and mean pore diameter were noticed when the Fe_3_O_4_-HBC composite was calcined (Table 1). But in this study, when the newly formed fayalite (BET: 6.03 (m^2^ g^−1^)) was added into arsenic containing solutions, it resulted in the formation of a highly reactive iron species ( = FeOOH). Therefore, the adsorbent mineralogical compositions played more important role for the removal of arsenic from aqueous solutions. In contrast, Fe_3_O_4_-HBC composite calcined at lower temperature (400°C) and oxygenated environment did not produce any reactive sites. Additionally, the surface area was also reduced to 0.95 m^2^ g^−1^ that further limited its removal performance. In aqueous medium, FeOOH (or hydrous ferric oxide, HFO) as a predominant immobilized species on iron surface reacted with As(III) and As(V) through ligand exchange by forming Fe-As complexes, which was also shown in a recent study [23].

On the other hand, due to higher chemical potential of oxygen at elevated calcination temperature (1000°C) under air, the fayalite formed quartz and magnetite/hematite [24] (Fig. 1d) with apparent XRD peaks (Fig. 1d). Moreover, the absence of C-H stretching bonding in the FTIR spectra (Fig. 1d) revealed complete decomposition of organic materials. Consequently, it was found that the resulted bare Fe_3_O_4_ had lower As(V) and As(III) removal efficiencies, which is in agreement with the results reported earlier [7], [16]. For example, Tian et al. [16] confirmed that the As(V) removal efficiency using bare Fe_3_O_4_ was only 6.98 mg g^−1^ as compared to the wheat straw loaded with Fe_3_O_4_ (30.34 mg g^−1^). Thus, they concluded that higher Fe_3_O_4_ content resulted to a higher arsenic adsorption capacity.

## Conclusions

To summarize, in current work, the synthesized Fe_3_O_4_-HBC composite was calcined at 400°C and 1000°C under air and nitrogen. High temperature calcination promoted phase transformation and formed a new iron silicate phase (fayalite, Fe_2_SiO_4_), which in turn generated highly reactive site ( = FeOOH) in aqueous system. Thus, Fe_3_O_4_-HBC-1000°C(N_2_) showed higher removal efficiency for both As(V) and As(III) at a wider pH range (4–10) as compared to other adsorbent composites. The adsorption of As(V) and As(III) is an endothermic process and maximum adsorption capacity of adsorbent composites increases from 25°C to 45°C. The presence of elevated concentration of phosphate was found to strongly inhibit As(V) and As(III) adsorption. Furthermore, the possible adsorption mechanism was demonstrated. This study suggests that the selective calcination process can be useful to improve the adsorbent efficiency for enhanced arsenic removal from contaminated aqueous environments.

## Supporting Information

Figure S1 **HT-XRD patterns of the adsorbent composite (Fe_3_O_4_-HBC-1000°C(N_2_) heated at 1000°C. Different HT-XRD peaks are also marked.**
(TIF)

Figure S2 **The variations in final or equilibrium pH of all the five adsorbents to remove As(V) (a) and As(III) (b) as a function of initial pH. (Experiment conditions: adsorbent dose  =  0.02 g 100 mL^−1^, temperature  = 25±0.8°C, agitation speed  = 150 rpm, initial concentration  = 100 µg L^−1^, contact time  = 14 h).**
(TIF)

Figure S3 **(A and B) Pseudo-first-order (PFO) kinetics model for As(V) and As(III), (C and D) Pseudo-second-order (PSO) kinetics model of the adsorbent: a) Fe_3_O_4_-HBC uncalcined, b) 400 °C under air, c) 400 °C under nitrogen, d) 1000 °C under air, e) 1000 °C under nitrogen (Experiment conditions: initial concentraion  = 100 µg L^−1^, adsorbent dose  = 0.02 g 100 mL^−1^, pH 7, temperature  = 25±0.8°C).**
(TIF)

Table S1 **Percentage (%) removal of As(V) and As(III) at different temperatures on calcined and uncalcined adsorbent composite. (Experimental conditions: adsorbent dose  = 0.02 g 100 mL^−1^, pH = 7.0, agitation speed  = 150 rpm, initial concentration  = 100 µg L^−1^, contact time  = 14 h).**
(DOCX)
