# Supplementary figures and images for: Arsenic Removal from Aqueous Solutions Using Fe3O4-HBC Composite: Effect of Calcination on Adsorbents Performance
Source: PLoS One. 2014 Jun 26;9(6):e100704. doi: 10.1371/journal.pone.0100704 (PMC4072660; doi:10.1371/journal.pone.0100704)

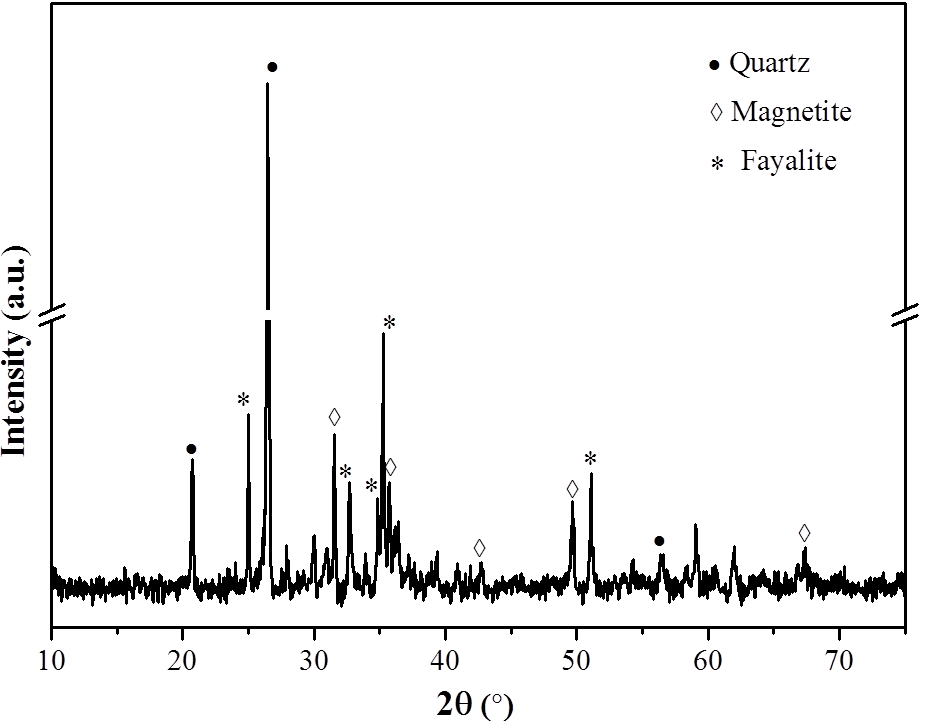

Supplement: Figure S1 — HT-XRD patterns of the adsorbent composite (Fe3O4-HBC-1000°C(N2) heated at 1000°C. Different HT-XRD peaks are also marked. (TIF) [file pone.0100704.s001.tif]

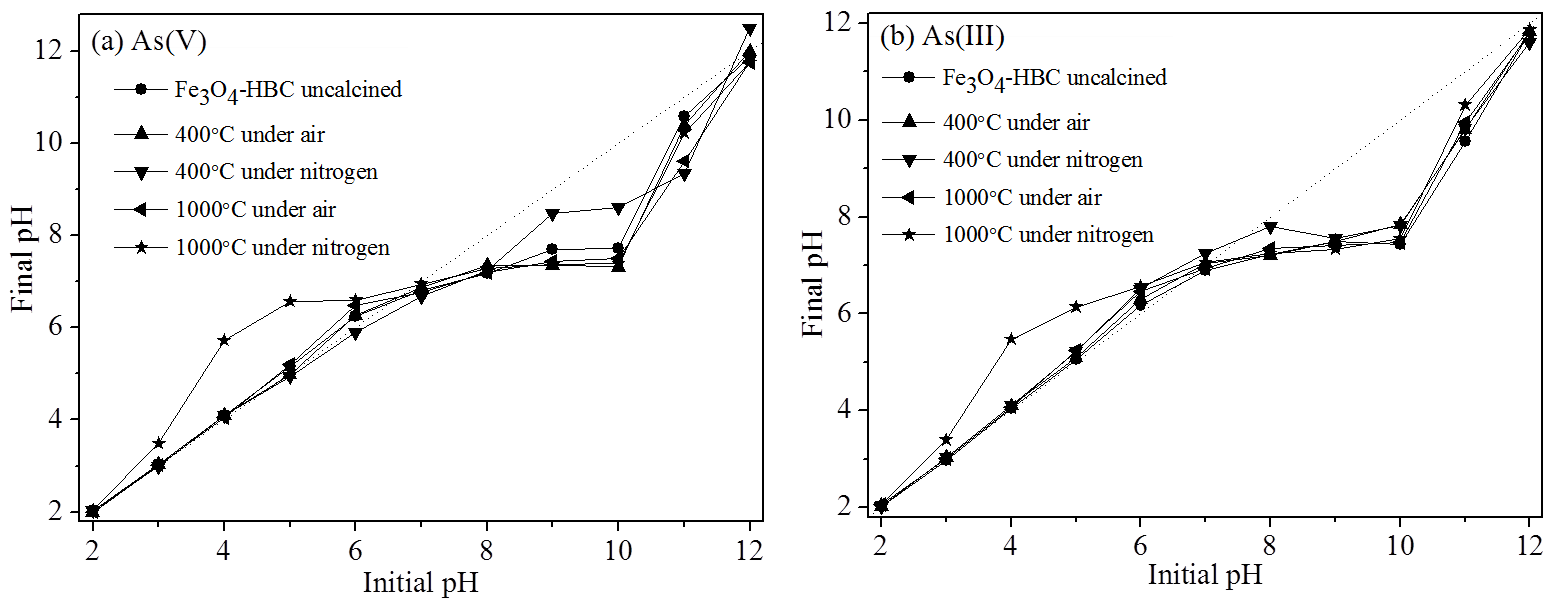

Supplement: Figure S2 — The variations in final or equilibrium pH of all the five adsorbents to remove As(V) (a) and As(III) (b) as a function of initial pH. (Experiment conditions: adsorbent dose = 0.02 g 100 mL−1, temperature = 25±0.8°C, agitation speed = 150 rpm, initial concentration = 100 µg L−1, contact time = 14 h). (TIF) [file pone.0100704.s002.tif]

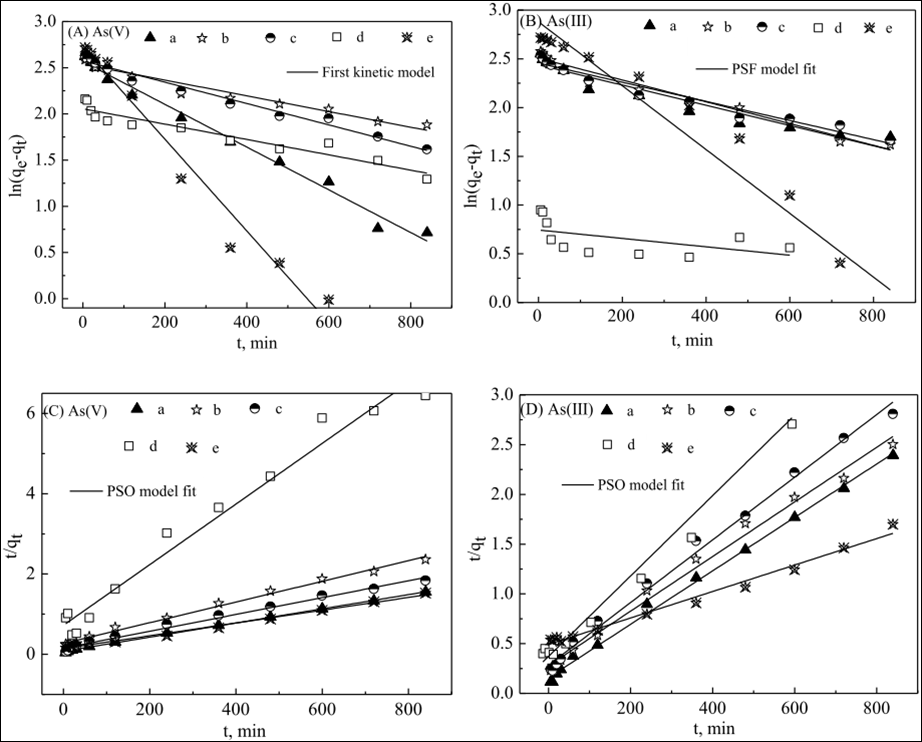

Supplement: Figure S3 — (A and B) Pseudo-first-order (PFO) kinetics model for As(V) and As(III), (C and D) Pseudo-second-order (PSO) kinetics model of the adsorbent: a) Fe3O4-HBC uncalcined, b) 400 °C under air, c) 400 °C under nitrogen, d) 1000 °C under air, e) 1000 °C under nitrogen (Experiment conditions: initial concentraion = 100 µg L−1, adsorbent dose = 0.02 g 100 mL−1, pH 7, temperature = 25±0.8°C). (TIF) [file pone.0100704.s003.tif]
