# Supplementary material for: Arsenic Removal from Aqueous Solutions Using Fe3O4-HBC Composite: Effect of Calcination on Adsorbents Performance
Source: PLoS One. 2014 Jun 26;9(6):e100704. doi: 10.1371/journal.pone.0100704 (PMC4072660; doi:10.1371/journal.pone.0100704)
Supplement: Table S1 — Percentage (%) removal of As(V) and As(III) at different temperatures on calcined and uncalcined adsorbent composite. (Experimental conditions: adsorbent dose = 0.02 g 100 mL−1, pH = 7.0, agitation speed = 150 rpm, initial concentration = 100 µg L−1, contact time = 14 h). (DOCX) [file pone.0100704.s004.docx]

| Adsorbent | Arsenic species | Percentage (%) removal at different temperature | | |
| --- | --- | --- | --- | --- |
|  |  | 25°C | 35°C | 45°C |
| Fe_3_O_4_-HBC uncalcined | As(V) | 98.2±0.1 | 98.7±0.2 | 99.0±0.6 |
| 400°C under air | As(V) | 89.1±0.2 | 90.0±0.3 | 89.7±0.8 |
| 400°C under nitrogen | As(V) | 88.7±0.1 | 90.7±0.2 | 91.7±0.3 |
| 1000°C under air | As(V) | 29.9±0.1 | 34.2±0.2 | 34.4±0.5 |
| 1000°C under nitrogen | As(V) | 100±0.0 | 100±0.0 | 100±0.0 |
| Fe_3_O_4_-HBC uncalcined | As(III) | 85.5±1.3 | 87.2±0.7 | 90.1±1.2 |
| 400°C under air | As(III) | 87.7±7.5 | 88.3±0.4 | 87.8±1.5 |
| 400°C under nitrogen | As(III) | 81.1±0.4 | 81.7±1.2 | 84.6±1.0 |
| 1000°C under air | As(III) | 09.7±0.1 | 11.3±1.0 | 12.7±0.2 |
| 1000°C under nitrogen | As(III) | 99.4±0.1 | 99.6±0.3 | 99.5±0.5 |
